# Genome Sequences of New Faustovirus Strains ST1 and LC9, Isolated from the South of France

**DOI:** 10.1128/genomeA.00613-17

**Published:** 2017-07-13

**Authors:** Amina Cherif Louazani, Julien Andreani, Maryem Ouarhache, Sarah Aherfi, Emeline Baptiste, Anthony Levasseur, Bernard La Scola

**Affiliations:** URMITE, Aix Marseille Université, UM63, CNRS 7278, IRD 198, INSERM 1095, IHU-Méditerranée Infection, Marseille, France

## Abstract

Faustoviruses are amoeba-infecting giant viruses closely related to the *Asfarviridae* family. Here, we report the isolation, genome sequencing, and annotation of ST1 and LC9, two new strains belonging to lineages L and E9 of faustoviruses, currently represented by only one representative each.

## GENOME ANNOUNCEMENT

Faustoviruses are an expanding proposed family of nucleocytoplasmic large DNA viruses, or amoeba-infecting giant viruses, isolated on *Vermamoeba vermiformis* (1). Analyses of their genomes have revealed homology with *Asfarviridae* and kaumoebavirus, suggesting a shared origin (2, 3). Here, we report the complete genome sequence of faustovirus strain ST1 and the draft genome annotation of faustovirus strain LC9. Faustovirus ST1 was isolated from wastewater in Saint-Pierre-de-Mézoargues, France, and strain LC9 was isolated from sewage in La Ciotat, France.

ST1 was isolated following the procedure previously described by Bou Khalil et al. (4). It was produced and purified for genome sequencing on the Illumina MiSeq platform with a 2 × 250-bp library in paired-end, parallel format. The genome was assembled with the Abyss program (5) and joined into a single scaffold with SSPACE (6). The LC9 draft genome sequences were retrieved from the European Nucleotide Archive database (WGS Sequence Set no. CZDJ01000000).

Subsequently, the analysis of both genomes followed the same procedure. tRNAs were predicted using tRNAscan-SE (7) and ARAGORN (8) software. Protein-coding genes were predicted using GenemarkS (9). Predicted proteins above 50 amino acids in length and presenting at least one best hit in a BLASTp search against the NCBI nonredundant protein sequence database (E-value > 1 × 10^−3^), or above 99 amino acids in length and no BLASTp best hit, were considered for further analysis. For functional annotation, predicted proteins were searched for conserved domains and putative functions using the Pfam protein families database (10) and NCBI Conserved Domain Database search tools (11). For phylogenetic analysis, amino acid sequences were aligned using the MUSCLE program (12). The maximum likelihood statistical method with 1,000 bootstrap replicates using the Jones-Taylor-Thornton (JTT) model for amino acid substitution was used for tree construction on MEGA7 software (13).

Faustovirus ST1 has a total genome size of 470,659 bp and displays a G+C content of 36.7%. The five scaffolds comprising the faustovirus LC9 genome have a total size of 470,227 bp and a mean G+C content of 39.8%. Results of tRNA prediction on both genomes showed the absence of tRNAs. The BLASTp and functional annotation resulted in 478 and 477 genes for ST1 and LC9, respectively. Among these genes, seven in ST1 and eight in LC9 present no homology on the NR database, constituting ORFans.

Phylogenetic analysis revealed that the new strains cluster with previously described lineages of faustovirus (L for ST1 and E9 for LC9) (3). It is important to note that the ST1 and LC9 strains represent the second isolates in these lineages, which were previously represented by only one strain each (faustovirus Liban for L and faustovirus E9 for E9). These results suggest that similar strains are spread in separate environments worldwide: faustovirus Liban being isolated from seawater in Lebanon and ST1 from house wastewater in France. This observation is in concordance with previously reported results for *Marseilleviridae* and *Mimiviridae* spp. (14–16). Moreover, faustoviruses are recurrently isolated from sewage and wastewater, suggesting the presence of a potential reservoir in close human proximity.

### Accession number(s).

The complete annotated genome sequences of faustovirus strains ST1 and LC9 have been deposited in ENA under the accession numbers LT839607 and CZDJ02000000, respectively.
